# Blue LED causes autophagic cell death in human osteosarcoma by increasing ROS generation and dephosphorylating EGFR

**DOI:** 10.1111/jcmm.16412

**Published:** 2021-05-07

**Authors:** Mingyu He, Gege Yan, Yang Wang, Rui Gong, Hong Lei, Shuting Yu, Xiaoqi He, Guanghui Li, Weijie Du, Tianshuai Ma, Manqi Gao, Meixi Yu, Shenzhen Liu, Zihang Xu, Elina Idiiatullina, Naufal Zagidullin, Valentin Pavlov, Benzhi Cai, Ye Yuan, Lei Yang

**Affiliations:** ^1^ Department of Pharmacology College of Pharmacy (The Key Laboratory of Cardiovascular Medicine Research, Ministry of Education) Harbin Medical University Harbin China; ^2^ Department of Orthopedics Department of Pharmacy The First Affiliated Hospital of Harbin Medical University The Second Affiliated Hospital of Harbin Medical University Harbin China; ^3^ Department of Clinical Pharmacology College of Pharmacy Harbin Medical University Harbin China; ^4^ Research Unit of Noninfectious Chronic Diseases in Frigid Zone Chinese Academy of Medical Sciences Harbin China; ^5^ Central Laboratory of Scientific Research Bashkir State Medical University Ufa Russia

**Keywords:** autophagy, beclin‐1, blue light‐emitting diodes (LED), cell death, epidermal growth factor receptor (EGFR), mitochondrial reactive oxygen species (ROS), osteosarcoma (OS)

## Abstract

Osteosarcoma (OS) is the most common primary malignant bone tumour in adolescence. Lately, light‐emitting diodes (LED)‐based therapy has emerged as a new promising approach for several diseases. However, it remains unknown in human OS. Here, we found that the blue LED irradiation significantly suppressed the proliferation, migration and invasion of human OS cells, while we observed blue LED irradiation increased ROS production through increased NADPH oxidase enzymes NOX2 and NOX4, as well as decreased Catalase (CAT) expression levels. Furthermore, we revealed blue LED irradiation‐induced autophagy characterized by alterations in autophagy protein markers including Beclin‐1, LC3‐II/LC3‐I and P62. Moreover, we demonstrated an enhanced autophagic flux. The blockage of autophagy displayed a remarkable attenuation of anti‐tumour activities of blue LED irradiation. Next, ROS scavenger N‐acetyl‐L‐cysteine (NAC) and NOX inhibitor diphenyleneiodonium (DPI) blocked suppression of OS cell growth, indicating that ROS accumulation might play an essential role in blue LED‐induced autophagic OS cell death. Additionally, we observed blue LED irradiation decreased EGFR activation (phosphorylation), which in turn led to Beclin‐1 release and subsequent autophagy activation in OS cells. Analysis of EGFR colocalization with Beclin‐1 and EGFR‐immunoprecipitation (IP) assay further revealed the decreased interaction of EGFR and Beclin‐1 upon blue LED irradiation in OS cells. In addition, Beclin‐1 down‐regulation abolished the effects of blue LED irradiation on OS cells. Collectively, we concluded that blue LED irradiation exhibited anti‐tumour effects on OS by triggering ROS and EGFR/Beclin‐1‐mediated autophagy signalling pathway, representing a potential approach for human OS treatment.

## 1 INTRODUCTION

Osteosarcoma (OS), derived from primitive mesenchymal cells, is the most common primary solid malignancy of bone, which is specifically most prevalent in teenagers and young adults.^1^, ^2^ In general, therapeutic intervention of OS involves a combination of treatments including surgery, chemotherapy and radiation therapy.^3^ The survival rate of OS patients has increased considerably due to the advanced treatment strategies.^4^ However, all or part of an arm/leg may have to be removed/amputated to ensure that all cancer gone in some cases. Thus, new and complex challenges regarding the safe therapy of OS patients continue to rise.

It is becoming increasingly apparent that increased mitochondrial reactive oxygen species (ROS) production has been observed to be a hallmark of many tumours.^5^ Accumulated evidence suggested ROS‐induced autophagy regulates cell apoptosis, proliferation and differentiation in multiple diseases.^6^ Additionally, it has been reported that in most contexts, autophagy facilitates tumorigenesis, but in some contexts, autophagy suppresses tumorigenesis.^7^ Pre‐clinical and ongoing clinical trials also showed autophagy plays a critical role in cancer therapies.^8^ Autophagy, as an adaptive response to stress, has been associated with both cell survival and cell death.^9^ Commonly, cancer cells display dysregulation of autophagy as compared to the normal cells, which results in malignant transformation and poor prognosis of cancer.^10^ Moreover, the cross‐talk between epidermal growth factor receptor (EGFR)‐mediated autophagy and cell death has also been currently utilized for cancer treatment.^11^ It has been shown that EGFR signalling negatively regulates autophagic cell death by inhibiting key autophagy factors such as Beclin‐1,^12^ which promotes formation of Beclin‐1‐Vps34‐Vps15 complexes and thereby induces autophagy.^13^ Several studies have also demonstrated the therapeutic effects of some components on OS through autophagy induction.^14^, ^15^ Therefore, autophagy is a critical mechanism for improving OS therapy.

Light‐emitting diodes (LEDs)‐based therapy/phototherapy has been widely reported their use in several aspects including acne, rosacea, ageing and wound healing.^16^, ^17^, ^18^, ^19^ The majority studies, however, were performed in the red or infrared spectrum. However, so far whether blue LED therapy is an effective treatment for OS has not been elucidated. A study by Chang et al. demonstrated that blue light exposure can trigger PI3K signalling activation and regulate neurite outgrowth and filopodia formation in rat hippocampal neurons by stimulation of receptor tyrosine kinases (RTKs).^20^ Interestingly EGFR, belonging to the ErbB family of RTKs,^21^ has been demonstrated a known hallmark for multiple human carcinomas.^22^ Hence, we assumed that EGFR might be sensitive under blue LED irradiation. Therefore, the objective of this study was for the first time to evaluate the effect of blue LED irradiation on human OS, and then determine the mechanisms involved in both cell apoptosis and autophagy induced by blue LED irradiation.

## 2 MATERIALS AND METHODS

### 2.1 Cell culture

Human osteosarcoma U‐2 OS (ATCC® HTB‐96™) and 143B cells were cultured in Dulbecco`s modified Eagle medium (DMEM) (Life Technologies Corporation, California, United States) and RPMI Medium 1640 basic medium (Thermo Fisher Scientific, Massachusetts, United States), containing 4500 mg/L glucose supplemented with 10% foetal bovine serum (FBS) (Biological Industries, Israel) and at 37℃ in an atmosphere containing 5% CO_2_. 10 μM Diphenyleneiodonium (DPI, MCE, HY‐100965) was added to the cells an hour before irradiation.

### 2.2 LED irradiation

Cells were irradiated at room temperature with red (peaked at 630 nm) / green (peaked at 560 nm) / blue (peaked at 470 nm) LED lights under the power density of 100 mW/cm^2^ for 180 J/cm^2^ (height: 9 cm, irradiation time: 30 minutes), 360 J/cm^2^ (height: 9 cm, irradiation time: 1 hour), 720 J/cm^2^ (height: 9 cm, irradiation time: 2 hours) and 1080 J/cm^2^ (height: 9 cm, irradiation time: 3 hours), respectively.

### 2.3 Trypan blue staining and cell counting assay

Ten μl of 0.4% Trypan Blue solution (LEAGENE, Beijing, China) in PBS was added in the tube containing suspended cells, after irritations with red/green/blue LED lights or treated the cells with 10 mM N‐acetyl‐L‐cysteine (NAC, A105422, aladdin, Shanghai, China) for 2 hours. After 5 minutes incubation, cells were counted using count star easy cell analysis (Count star, Shang Hai, China).

### 2.4 Propidium iodide (PI)/Hoechst 33342 staining

PI/Hoechst 33342 staining was performed using Hoechst 33342/PI Double Staining Kit (Solarbio Science, Beijing China). Cells were plated into a glass bottom cell culture dish at the density of 2.0 × 10^5^. Briefly, the cells were fixed with 4% paraformaldehyde for 15 minutes at 37℃. The stain solution was added into cell culture medium for 30 minutes at room temperature in the dark. Finally, images of the staining were captured by the confocal laser scanning microscope (FV10i, Olympus, Tokyo, Japan).

### 2.5 Ethynyl‐2‐deoxyuridine (EdU) cell proliferation assay

The assay was described previously^23^ using EdU Apollo DNA in vitro kit (Ribobio, Guangzhou, China). Cells were plated into a glass bottom cell culture dish (NEST, Hong Kong, China) at the density of 2.0 × 10^5^. Briefly, cells were fixed with 4% paraformaldehyde (m/v) for 30 minutes, and followed by incubation of 30 μM EdU at 37℃ for 90 minutes. After permeabilized in 0.5% Triton X‐100, The Apollo staining solution was added into cell culture medium for 30 minutes in the dark. Finally, the cells were incubated with 20 μg/mL 4’,6‐diamidino‐2‐phenylindole (DAPI) for 10 minutes. The EdU index (%) was the average ratio of the number of EdU‐positive cells over total cells in five randomly selected areas under the confocal laser scanning microscope (FV10i).

### 2.6 Migration assays

Cells were plated into 6‐well culture plates at the density of 2.5 × 10^5^ cells/mL. When the confluence of cells reached to 70%, the cells were starved in DMEM (contained with 0.4% FBS) for 12 hours. After starvation, a wound was created by scraping the cells with a 200 μL pipette tip. Cells were washed with PBS for twice and cultured in DMED (contained with 0.4% FBS) for 72 hours. After that, cells were treated with blue LED irradiation. Images were captured for long‐term monitoring at 0/24/48/72 hours and for short‐term monitoring at 0/6/12/24 hours after wounding with a standard light microscopy (ECLIPSE TS100, Nikon, Japan). The wound area was measured using ImageJ software (National Institutes of Health (NIH), United States).

### 2.7 Invasion assays

A 24 mm Transwell® chambers with precoated Matrigel (Corning #3422, United States) was used to detect cell invasive abilities according to the manufacturer’s protocol. After starvation of cells for 12 hours, cells at the density of 5 × 10^4^ cells/mL in serum‐free DMEM medium were seeded in the upper chamber. DMEM medium contained with 10% FBS was added into the lower chamber. The cells were then irradiated with blue LED at a power density of 100 mW/cm^2^. After 24 hours, cells migrated through the membrane were stained with 0.1% crystal violet (Beyotime Biotechnology, China) for 15 minutes and counted using a light microscopy (ECLIPSE TS100, Nikon).

### 2.8 Live‐cell imaging for autophagic flux

The mRFP‐GFP‐LC3 adenoviral particles were purchased from HANBIO (Shanghai, China). MRFP was used to flag and track LC3. The weakening of GFP indicated the fusion of lysosomes and autophagosomes to form autophagic lysosomes. GFP fluorescent protein is sensitive to acidity. When autophagosomes and lysosomes fused, GFP fluorescence will be quenched, but red fluorescence can be detected. 4 × 10^5^ U‐2 OS cells were infected with adenoviral particles; 6 hours post‐transfection, cells were washed with DMEM and kept culturing for another 48 hours. After that, imaging was performed immediately on a confocal laser scanning microscope (FV10i).

### 2.9 MitoSOX™ Red staining

The MitoSOX™ mitochondrial superoxide indicator (M36008, Thermo Fisher, USA) was dissolved with 13 μL dimethylsulphoxide (DMSO) to make a 5 mM MitoSOX™ reagent stock solution. The cells were incubated with 5 μM MitoSOX™ reagent working solution for 10 minutes at 37℃ and protected from light. Washed the cells gently three times with PBS and then fixed with 4% paraformaldehyde (m/v) for 30 minutes. Finally, the cells were incubated with 20 μg/mL 4’,6‐diamidino‐2‐phenylindole (DAPI) for 10 minutes. The fluorescence intensity was measured using ImageJ software (National Institutes of Health (NIH), United States).

### 2.10 Electron microscopy

For electron microscopy analysis, U‐2 OS cells were treated with blue LED for 360 J/cm^2^ and then centrifuged at 3000 r/min for 20 minutes. The cells were fixed immediately with 2.5% glutaraldehyde buffer (pH 7.4) at 4℃ overnight. Briefly, the samples were post‐fixed in 1% osmium tetroxide at room temperature for 1 hour, dehydrated in graded ethyl alcohol and then embedded in epon. After stained in saturated uranyl acetate and lead citrate, the cells were examined with a transmission electron microscope (HITACHI 7650, Tokyo, Japan) operated at 80 KV.

### 2.11 Cell transfection of small interfering RNA (siRNA)

U‐2 OS cells were seeded into a 6‐well plate at 2 × 10^5^ cells per well and cultured overnight. Beclin‐1 siRNA (sense: 5`‐GGTCTAAGACGTCCAACAATT‐3`; antisense: 5`‐TTGTTGGACGTCTTAGACCTT‐3`); negative control (NC) siRNA (sense: 5`‐TTCTCCGAACGTGTCACGTTT‐3`; antisense: 5`‐ACGTGACACGTTCGGAGAATT‐3`) Atg7 siRNA (sense: 5`‐GGAGTCACAGCTCTTCCTTTT‐3`; antisense: 5`‐AAGGAAGAGCTGTGACTCCTT‐3`) were transfected into cells using Lipofectamine 3000 reagent (Life Technologies Corporation) according to manufacturer’s protocol. Cells were harvested 48 hours after transfection.

### 2.12 Quantitative real‐time polymerase chain reaction (qRT‐PCR)

Total RNA was extracted using TRIzol reagent (Life technologies Corporation) followed the manufacturer`s protocol. 500 ng total RNA was reverse‐transcribed into cDNA in a total reaction volume of 10 μL by High Capacity cDNA Reverse Transcription Kit (Thermo Fisher Scientific, Waltham, Massachusetts, United States). Quantitative real‐time PCR analysis was performed with 1 μL cDNA using SYBR Green PCR Master (Roche) in a 7500 Fast Real‐Time instrument (Applied Biosystems, Foster City, CA, United States). Gene expression was normalized to endogenous GAPDH mRNA. The primers sequences of Beclin‐1 and GAPDH are as following: Beclin‐1‐Forward primer (F): 5`‐GACAGTGAACAGTTACAGATGG‐3`, Beclin‐1‐Reverse primer (R): 5`‐TCAGCCTGGACCTTCTCG‐3`; GAPDH‐F: 5`‐CATGTTCGTCATGGGTGTGAA‐3`, GAPDH‐R:GGCATGGACTGTGGTCATGAG.

### 2.13 Immunoprecipitation and Western blot

Cells were seed in 100 cm^2^ dishes and treated with blue LED. Washed the cells with cold PBS for 2 times immediately. Cells were collected with lysis buffer and centrifuged at 15000 rpm for 15 minutes at 4℃. Mixed the supernatants with protein A+G beads for 4 hours at 4℃ and then incubated with 4 μg antibody and 300 μg protein for 10 hours at 4℃. Washed the beads for 5 times and eluted. Subjected the beads to Western blot. The Western blot analysis was described previously.^24^ Briefly, cells were lysed in RIPA buffer (Beyotime Biotechnology) on ice for 1 hour. Protein fractions were collected by centrifugation at 13 500 rpm for 15 minutes, and then supernatants were heated with SDS buffer at 95℃ for 7 minutes. Proteins were subjected to SDS‐PAGE and transferred onto nitrocellulose membranes. After blocked with 5% fat‐free milk and incubated with specific antibodies overnight, the membranes were subjected to secondary antibodies at room temperature for 1 hours. Odyssey v1.2 software (LI‐COR Biosciences, Lincoln, NE, United States) was used to visualize the proteins. The following primary antibodies and concentrations were used: Beclin‐1 (1:1000, cat. no. 3738S, CST), LC3 (1:1000, cat. no. L7543, Sigma‐Aldrich, St. Louis, Missouri, United States), p62 (1:1000, cat. no. 5114S, CST), EGFR (1:500, cat. no. Bs‐10007R, BIOSS, Bioss Antibodies, Boston, Massachusetts, United States), pEGFR (Tyr1172, 1:500, cat. no. Bs‐20385R, BIOSS), NOX2 (1:1000, cat. no. ab129068, Abcam), NOX4 (1:1000, cat. no. 14347‐1‐AP, Proteintech), Catalase (CAT, 1:2000, cat. no. ab209211, Abcam) and β‐actin (1:1000, cat. no. 8457S, CST). All antibodies were diluted in phosphate‐buffered saline (PBS).

### 2.14 Immunofluorescence

Cells were plated into a glass bottom cell culture dish at the density of 2.0×10^5^ and fixed with 4% paraformaldehyde at 37℃ for 15 minutes. After washing twice with PBS, cells were permeabilized with 0.3% Triton X‐100 (Sigma‐Aldrich) for 15 minutes. Then the cells were blocking with goat serum. Primary antibodies of EGFR (1:50 dilution, Santa Cruz Biotechnology, Dallas, Texas, United States), pEGFR and Beclin‐1 were diluted in PBS and incubated at 4℃ overnight, and the secondary antibody was incubated for 1 hours at room temperature. Images were acquired using a confocal microscope (FV10i).

### 2.15 Statistical analysis

All assays were repeated at least three times, and values were given as mean ± SEM. Statistical analysis was performed by one‐way analysis of variance (ANOVA) for more than two‐group comparisons and Student’s t test was used for two‐group comparisons (GraphPad Software Inc., San Diego, CA). P values < 0.05 were considered statistically significant.

## 3 RESULTS

### 3.1 Blue LED irradiation induces OS cell death

To determine if irradiations of LED in different wavelengths could affect OS cell growth, we examined relative proportions of live and dead cells in red (peaked at 630 nm) / green (peaked at 560 nm) / blue (peaked at 470 nm) LED irradiated or non‐irradiated U‐2 OS cells. Trypan blue staining showed that red and green LED irradiation had no effects on U‐2 OS cell growth. The blue LED irradiation, however, displayed shrinkage and rounding of the cells under light microscope (Figure S1A), and caused a dramatical reduction in number of live cells as compared with untreated control groups (Figure S1B). Moreover, we observed blue LED treatment for more than irradiation dose of 360 J/cm^2^ hardly showed no more effective inhibitions on cell viability. In line with results observed above, Hoechst 33342 PI double staining indicated the percentages of dead cells were significantly increased after blue LED irradiation but not in red/green LED or non‐LED irradiated OS (Figure S1C and S1D). These data indicated that blue LED irradiation strongly inhibited cell growth and induced cell death in human OS.

### 3.2 Blue LED irradiation suppresses OS cell proliferation, migration and invasion

**FIGURE 1:**
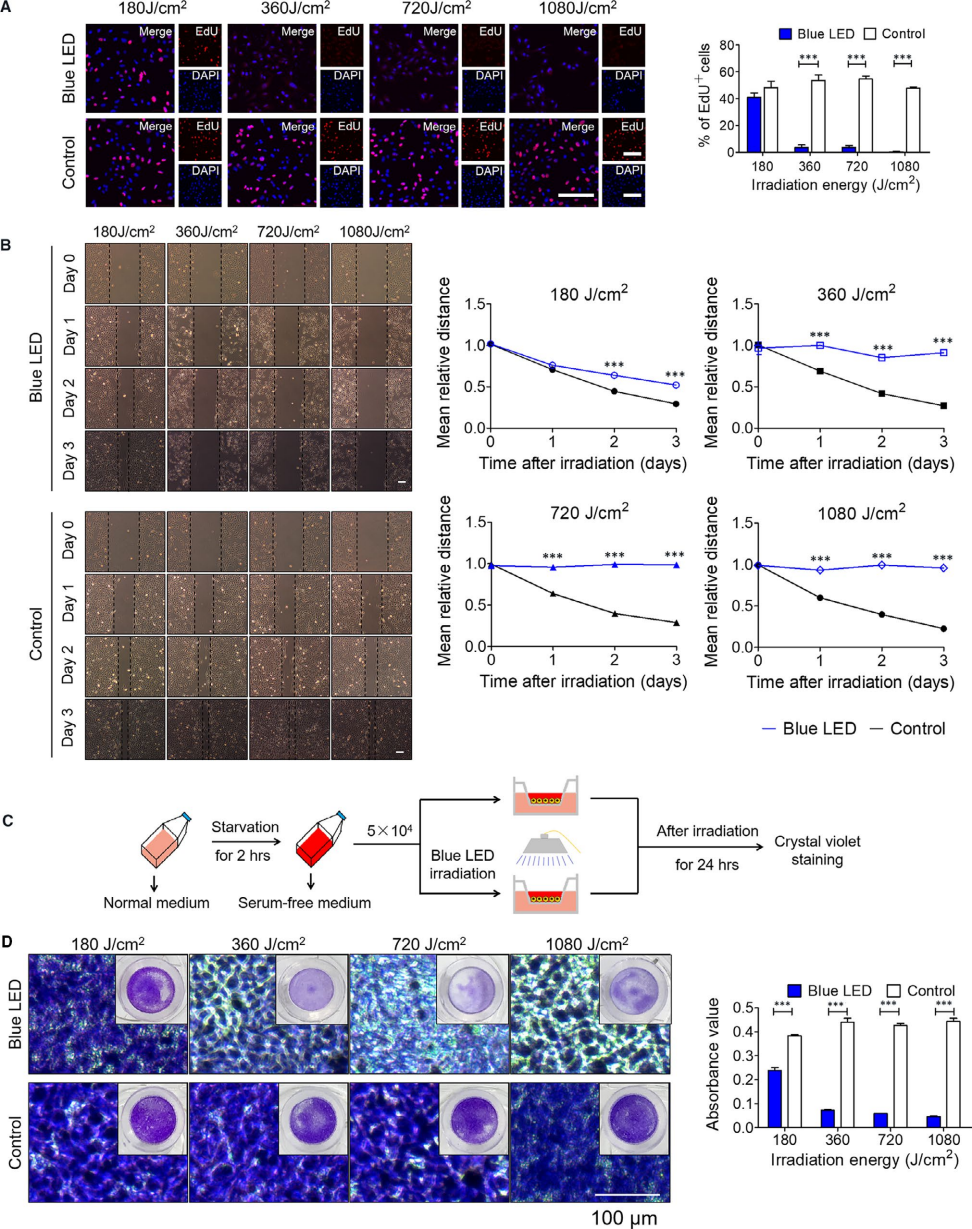
Blue LED irradiation restrains U‐2 OS cell proliferation, migration and invasion. U‐2 OS cells were irradiated with blue LED for 0 J/cm^2^, 180 J/cm^2^, 360 J/cm^2^, 720 J/cm^2^ and 1080 J/cm^2^ respectively. A, Cell proliferation was detected by EdU staining. (Bar: 100 μm) (left) The panels show percentages of the proliferative cells (EdU‐positive) (right). B, The migration was evaluated by Wound‐healing assay. (Bar: 200 μm) (left) The mean relative distance of migrated cells (right). C, Experimental flow chart of Trans‐well assay. D, The absorbance value of crystal violet at 570 nm reflects the cell invasion capacity. Data are expressed as the mean ± SEM. ***P < 0.001

To assess the effects of blue LED irradiation on cell proliferation, we used EdU staining assay in U‐2 OS cells upon different doses of blue LED irradiation for 180 J/cm^2^, 360 J/cm^2^, 720 J/cm^2^ and 1080 J/cm^2^ respectively. As shown in Figure 1A, the relative low irradiation doses of 180 J/cm^2^ failed to inhibit cell proliferation compared with non‐irradiated control cells. The cell proliferation was decreased from by 50% to 5% in OS cells starting from 360 J/cm^2^. Furthermore, wound‐healing and Trans‐well assays were then employed to evaluate whether cell invasion and migration were affected by blue LED irradiation in OS cells. As shown in Figure 1B, the wound‐healing assay revealed that the gap between the scorings was larger in blue LED irradiation for 180 J/cm^2^, 360 J/cm^2^, 720 J/cm^2^ and 1080 J/cm^2^ groups than in control group in 24, 48 and 72 hours. In addition, significant suppression of the migration potential of U‐2 OS cells was also observed at shorter time points (6 and 12 hours) after treatment with blue LED lights (Figure S2). Similar results were also observed in Trans‐well assays. Schematic representation of the Trans‐well workflow was shown in Figure 1C. The cell invasion through matrigel‐coated membranes was markedly inhibited in blue LED‐treated groups compared with control groups (Figure 1D). Overall, the migration rate of blue LED‐treated cells decreased to 23% and the invasion rate decreased to 10%. These results demonstrated that blue LED irradiation impaired cell proliferation, migration and invasion in human OS.

### 3.3 Blue LED irradiation induces ROS accumulation in OS cells

**FIGURE 2:**
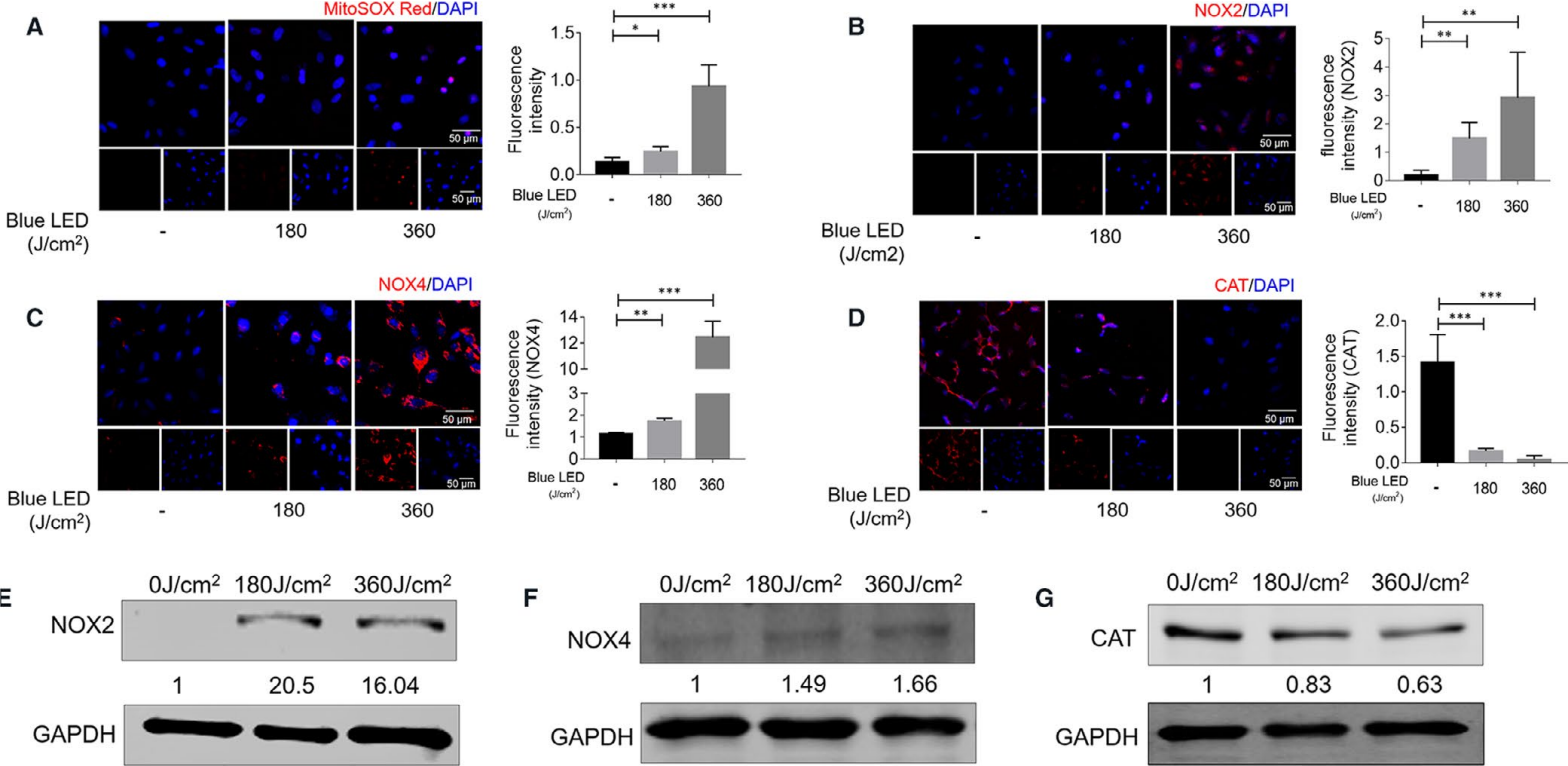
Blue LED irradiation increases mitochondrial reactive oxygen species (ROS) production in U‐2 OS cells. U‐2 OS cells were treated with blue LED irradiation for 0 J/cm^2^, 180 J/cm^2^ and 360 J/cm^2^ respectively. A, MitoSOX Red Indicator staining shows ROS in U‐2 OS after exposure to blue LED irradiation and the panels show the percentage of fluorescence intensity of ROS. (Bar: 50 μm) (B‐D) Immunofluorescence analysis of NOX2 (B), NOX4 (C) and CAT (D) in U‐2 OS. Nuclei were stained with DAPI. The panels show the percentage of fluorescence intensity of NOX2, NOX4 and CAT. Scale bars, 50 μm. (E‐G) Western blot assays show the protein levels of NOX2 (E), NOX4 (F) and CAT (G) in U‐2 OS followed by exposure to blue LED irradiation. Data are expressed as the mean ± SEM. *P < 0.05; **P < 0.01; ***P < 0.001

We next investigated whether blue LED irradiation stimulates ROS production in OS cells. U‐2OS cells were treated with 180 J/cm^2^ and 360 J/cm^2^. MitoSOX‐based assay results showed that blue LED irradiation induces ROS accumulation in OS cells (Figure 2A). NADPH oxidase 2 (NOX2) and NADPH oxidase 4 (NOX4) are critical components of the phagocytic NADPH oxidase inducing ROS production.^25^ As shown in Figure 2B‐G, the protein expression levels of NOX2 and NOX4 were significantly increased, but under blue LED irradiations for 180J/cm^2^ and 360J/cm^2^ in U‐2 OS cells validated by both Western blotting analysis and immunofluorescence staining (IFS). In addition, catalase (CAT) is a very important enzyme in protecting the cell from oxidative damage by ROS. The results revealed that CAT protein levels were decreased under blue LED irradiations in U‐2 OS cells. These effects were further confirmed in another osteosarcoma cell line 143B. As shown in Figure S3, blue LED irradiations noticeably inhibited 143B cell growth and cell migration. And IFS revealed that the levels of NOX2 and NOX4 were significantly increased, but CAT decreased under blue LED irradiations for 360J/cm^2^ in 143B cells. Accordingly, blue LED irradiation induces ROS accumulation in OS cells. Next, TUNEL assay was performed to assess whether the implication of cell apoptosis under blue LED irradiation in osteosarcoma cells. We found that the percentages of apoptotic cells were markedly increased following treatment with blue LED irradiation for 360 J/cm^2^, 720 J/cm^2^ and 1080 J/cm^2^. Furthermore, inhibition of cell apoptosis by the general caspase inhibitor Z‐VAD‐FMK at least partially rescued U‐2 OS cell proliferation affected by blue LED irradiations (shown in Figure S4).

### 3.4 Blue LED irradiation induces autophagy in OS

**FIGURE 3:**
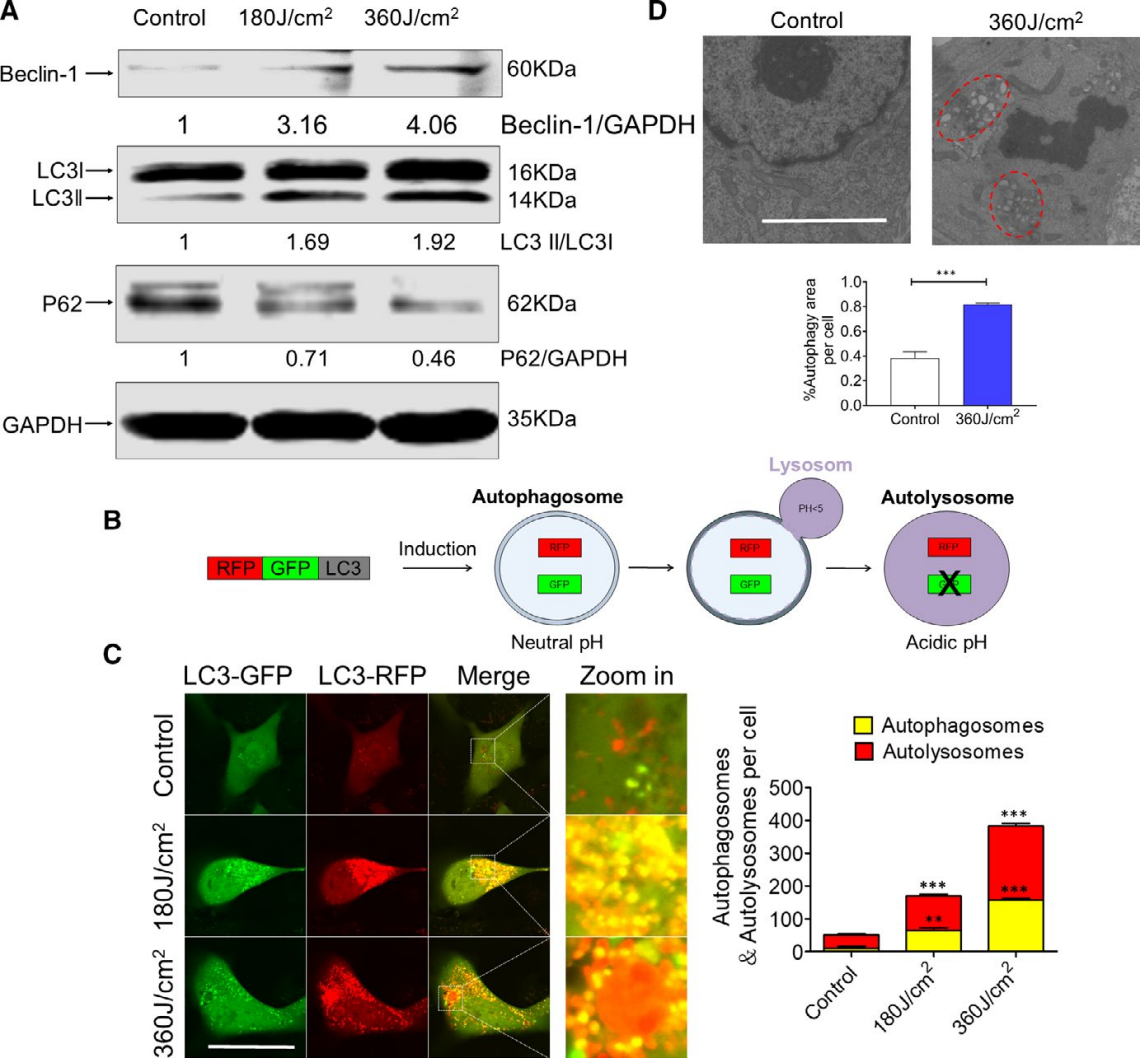
Blue LED irradiation induces U‐2 OS cell autophagy. U‐2 OS cells were firstly treated with blue LED light for 180 J/cm^2^ and 360 J/cm^2^. A, Western blot was used to detect the autophagy‐associated proteins levels including Beclin‐1, LC3 and p62, which were normalized to β‐actin. B, The basic principles of autophagic flu experiment. C, U‐2 OS cells infected with mRFP‐GFP‐LC3 virus, and the images were captured with a confocal microscopy after the blue LED irradiation for 180 J/cm^2^ and 360 J/cm^2^. Red dots represent autophagosomes while the red and green merged (yellow) dots represent autolysosomes. (Bar: 50 μm) Bar graph demonstrates that the number of autophagosomes and autolysosomes in U‐2 OS cells. D, Representative TEM images in U‐ 2 OS cells treated with or without blue LED irradiation for 360 J/cm^2^. Bar graph demonstrates the whole size of autophagosomes, lysosomes and autolysosomes. Data are expressed as the mean ± SEM. *P < 0.05; **P < 0.01; ***P < 0.001

Recent studies have described ROS can regulate autophagy through the regulation of autophagy gene expression such as Beclin‐1 or P62 in cancer cells.^26^, ^27^ We next aimed to explore whether autophagy was involved in cell death induced by blue LED irradiation in U‐2 OS cells. As shown in Figure 3A, blue LED treatments resulted in elevated protein levels of autophagic markers Beclin‐1 and the ratio of cellular MAP1LC3 (LC3)‐II to LC3‐I, as well as decreased levels of sequestosome 1 (SQSTM1/p62) in a dose‐dependent manner. To determine the role of blue LED irradiation on the autophagic flux, we infected cells with an adenovirus tandem fluorescent‐tagged LC3 (mRFP‐GFP‐LC3) (Figure 3B). More ectopically expressed mRFP‐GFP‐LC3 was observed as red (autolysosomes formation) and yellow (autophagosomes formation) speckles in the blue LED irradiated cells than in non‐treated cells (Figure 3C). We next performed transmission electron microscopy (TEM) to observe the morphology of cells after treatment with or without blue LED irradiation for 360 J/cm^2^. The results shown in Figure 3D describe the typical morphology of autophagy in blue LED irradiated cells, and the area of autophagic vesicles per cell was markedly increased compared with non‐treated group. In line with the results for U‐2 OS, we also see a significant increase in LC3‐II/I ratio, as well as decrease in P62 and pEGFR protein levels for 143B (Figure S5A and B). Together, these findings suggest that blue LED irradiation induces autophagy in human OS.

### 3.5 The anti‐tumour activity of blue LED irradiation was reversed by an autophagy inhibitor on OS

**FIGURE 4:**
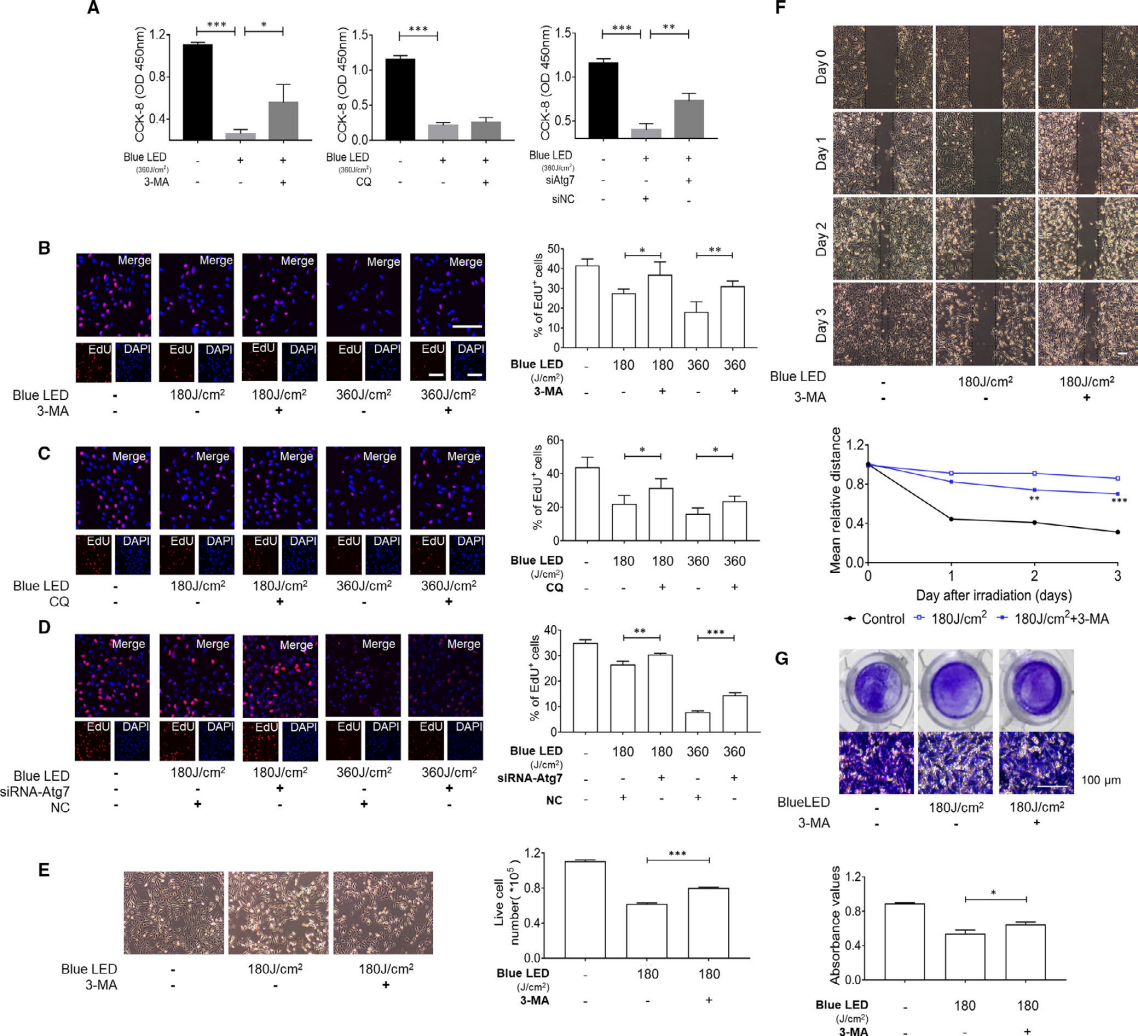
Blue LED irradiation hinders U‐2 OS cell growth mainly through induction of autophagy. CCK‐8 assays (A) and EdU staining (B‐D) were used to determine cell proliferation after exposure to blue LED irradiation and combined with treatment of 3‐MA (5 mM), chloroquine (CQ) and siRNA‐Atg7. (Bar: 100 μm) (E) Representative images acquired with a microscope (magnification ×4) after irradiation. (Bar: 200 μm) Bar graph demonstrates the number of live cells. F, Wound‐healing assay (Bar: 200 μm) and (G) Trans‐well assay exhibit migrated cells after treatment by 3‐MA combined with or without blue LED. Data are expressed as the mean ± SEM. *P < 0.05; **P < 0.01; ***P < 0.001

To further confirm the importance of autophagy in blue LED irradiation‐induced cell death, we treated U‐2 OS cells with an inhibitor of autophagy 3‐Methyladenine (3‐MA), chloroquine (CQ), or small interfering RNA (siRNA) against autophagy‐related gene 7 (Atg7) to block autophagosome formation.^28^ We found that cells treated with 3‐MA and siAtg7 were significantly resistant to the decreased cell activities caused by LED irradiations; however, cells treated with CQ were not (Figure 4A). Moreover, as shown in Figure 4B, the cells were irradiated with blue LED for 180 J/cm^2^ or 360 J/cm^2^ in the presence or absence of 3‐MA. Under this condition, EdU staining assay revealed that the number of proliferative cells was significantly increased in the presence of 3‐MA in blue LED irradiated cells. The percentages of EdU‐positive cells were 31.3 ± 3.2% versus 39.3 ± 3.6%, and 14.4 ± 4.5% versus 23.7 ± 5.1% in non‐treated and 3‐MA treated groups in 180 J/cm^2^ and 360 J/cm^2^ irradiation groups, respectively. Furthermore, inhibition of autophagy by CQ or siAtg7 partially recovered U‐2 OS cell proliferation inhibited by blue LED irradiation (Figure 4C and D). Moreover, consistent with the increased proliferative cells percentages, live cells number was significantly increased in comparison with blue LED treatment without 3‐MA (Figure 4E).

We next used Wound‐healing and Trans‐well assays to further validate the role of autophagy in blue LED irradiation‐induced inhibition of U‐2 OS cell migration and invasion. As shown in Figure 4F, the cells were treated with blue LED for 180 J/cm^2^ in the presence or absence of 3‐MA. We found that the migration abilities were significantly increased compared with blue LED treatment without 3‐MA. In line with the findings above, invasion abilities were also significantly increased in the group combination treatment with 3‐MA compared with blue LED irradiation alone group (Figure 4G). These results suggest that the suppressive effects of blue LED irradiation on human OS cells depend, at least partly, on its ability to lead to autophagy.

### 3.6 Blue LED irradiation leads to ROS‐induced autophagy on OS

Furthermore, we investigated whether ROS scavenger N‐acetyl cysteine (NAC) could recover blue LED irradiation‐mediated inhibition of cell proliferation, induction of cell death. As shown in Figure S6A, NAC attenuated ROS production by blue LED irradiation in U‐2 OS cells. In addition, EdU staining results showed that cell proliferation was recovered from 21.7% to 30.3%, from 2.5% to 19.6% at 5mM NAC in 180 J/cm^2^ and 360 J/cm^2^ irradiation groups, respectively (Figure S6B). Cell counting assay showed live cells number was also partly recovered at 5mM NAC in both 180 J/cm^2^ and 360 J/cm^2^ irradiation groups (Figure S6C). Next, we determined whether the ROS scavenger NAC can block blue LED‐mediated induction of autophagy. We treated U‐2 OS cells with blue LED irradiation combined with either treatments of NAC or not. As shown in Figure S6D, NAC treatments lead to an abolishment of blue LED irradiation‐induced autophagy. In line with these findings, the results revealed that NOX inhibitor DPI significantly increased OS cell proliferation and decreased ROS production in blue LED irradiation groups (Figure S7A and B). Furthermore, DPI blocked blue LED‐mediated induction of autophagy as well (Figure S7C).

### 3.7 Blue LED irradiation prevents EGFR activation (phosphorylation) and EGFR/Beclin‐1 complex formation

**FIGURE 5:**
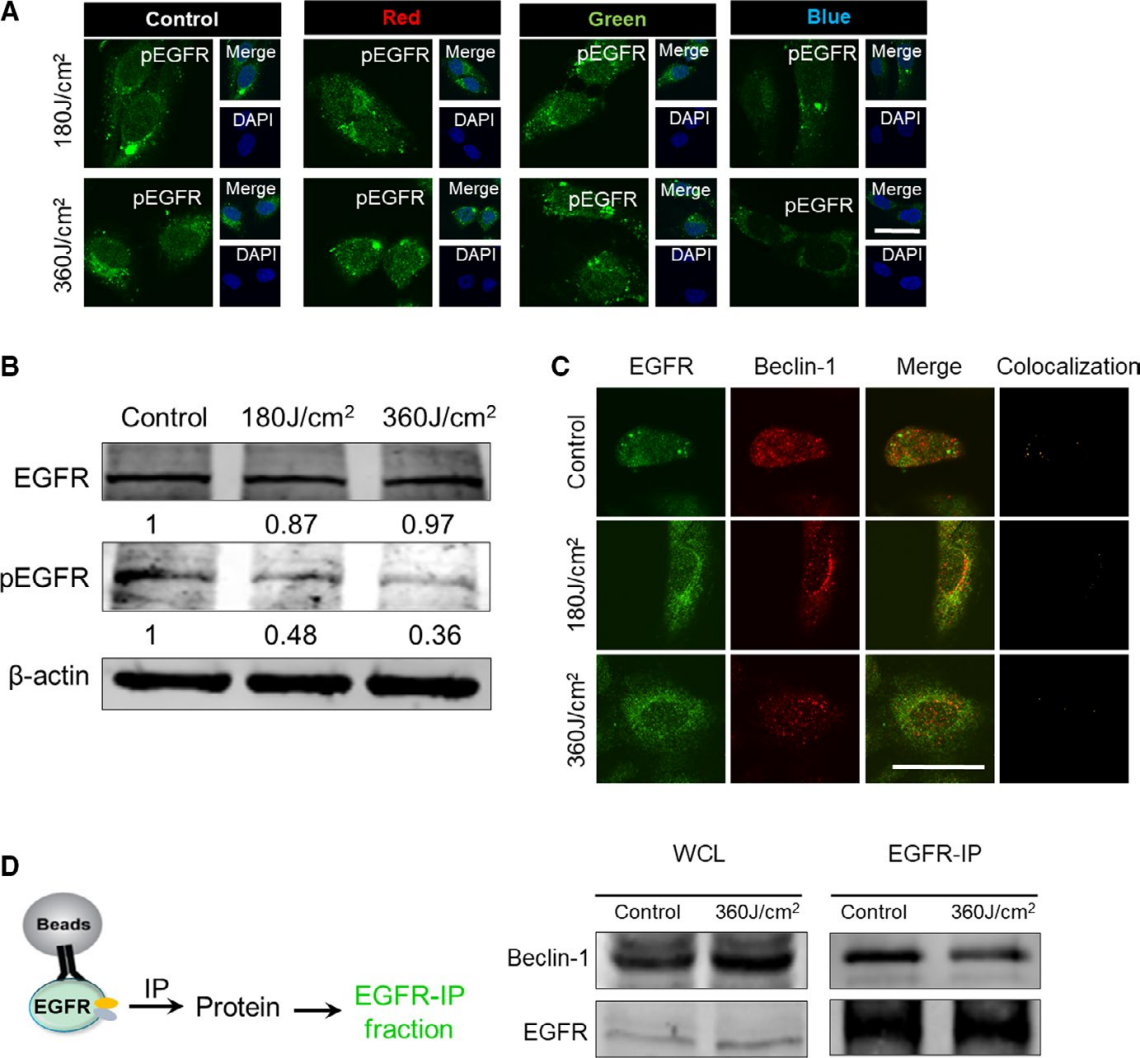
Blue LED irradiation prevents EGFR activation (phosphorylation) and EGFR/Beclin‐1 complex formation. U‐2 OS cells were subject to three types of red/green/blue LED irradiation for 180 J/cm^2^ and 360 J/cm^2^. A, The expression of pEGFR was detected by immunostaining promptly. Representative images were captured with a confocal microscopy. (Bar: 50 μm). B, The protein levels of total EGFR and pEGFR after treatment of blue LED. C, Colocalization of EGFR (Green) and Beclin‐1 (Red) was detected by immunostaining. The yellow spots indicate colocalization for both. (Bar: 50 μm). D, Validation of Beclin‐1 and EGFR combination in U‐2 OS cells detected by co‐immunoprecipitation (Co‐IP) with anti‐EGFR or anti‐IgG. Data are expressed as the mean ± SEM. **P < 0.01; ***P<0.001

Recent studies revealed that EGFR inhibition could trigger autophagy in cancers.^29^, ^30^ As an oncogenic receptor tyrosine kinase (RTK), EGFR was highly expressed in OS,^31^ which might be sensitive under blue light treatment.^20^ We therefore have been suggested that EGFR activation could be the target of blue LED irradiation on human OS. As shown in Figure 5A, immunofluorescence staining for phosphorylated EGFR (pEGFR) revealed that blue LED irradiation resulted in a dramatical decrease of pEGFR in a dose‐dependent manner in comparison with non‐irradiated group in both U‐2 OS and 143B osteosarcoma cells. However, red/green LED irradiation made almost no difference of pEGFR activities. Furthermore, Western blot analysis showed similar results in protein levels of pEGFR when cells were irradiated by blue LED lights for 180 J/cm^2^ and 360 J/cm^2^ (Figure 5B). Besides, EGFR activation (phosphorylation) has been proven to promote EGFR/Beclin‐1 complex formation and then autophagy inhibition.^32^ Thus, we next used confocal microscopy to examine colocalization of EGFR and Beclin‐1 in U‐2 OS cells after blue LED irradiation for 180 J/cm^2^ and 360 J/cm^2^. As expected, the results showed a strong decrease of colocalization indeed in cells after treatment by blue LED in comparison with non‐treated group (Figure 5C).

To further determine whether these two proteins, EGFR and Beclin‐1, directly interact, we performed a co‐immunoprecipitation (co‐IP) assay. After precipitation of EGFR protein by a specific antibody, Western blot assay was subsequently performed. As shown in Figure 5D, the results showed that Beclin‐1 was detected in EGFR‐IP group but almost disappeared in IgG‐IP and blue LED irradiation EGFR‐IP groups, indicating direct interaction between EGFR and Beclin‐1. Together, these data suggest that blue LED irradiation prevents EGFR activation (phosphorylation), followed by inhibition of EGFR/Beclin‐1 complex formation in U‐2 OS cells.

### 3.8 Blue LED irradiation triggers autophagy also via regulation of EGFR/Beclin‐1 pathway

**FIGURE 6:**
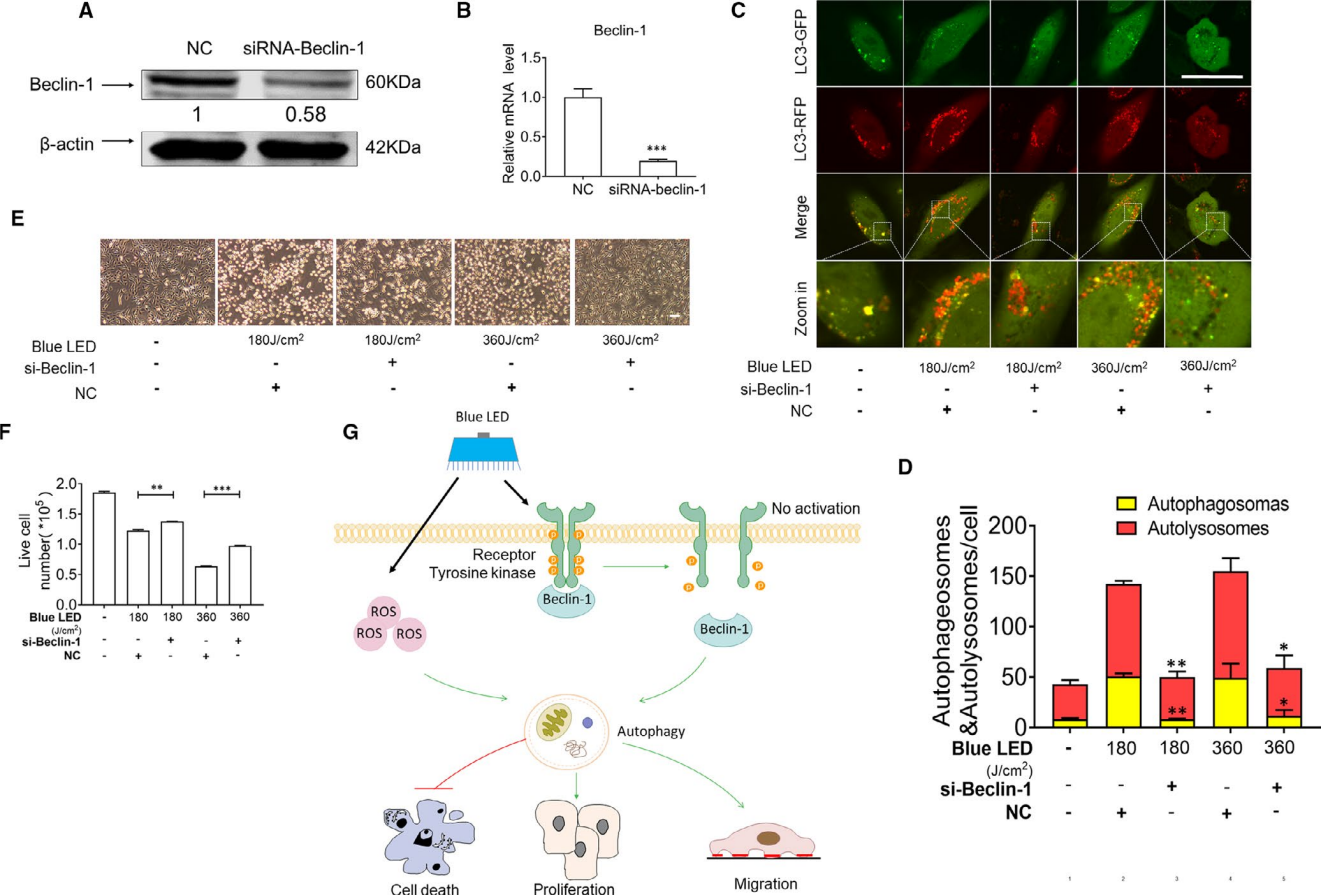
Blue LED irradiation triggers autophagy via regulation of EGFR/Beclin‐1 pathway. U‐2 OS cells were transfected with siRNA‐Beclin‐1. A, Western blot and B, qRT‐PCR validation of siRNA‐Beclin‐1 knockdown efficiency (C) U‐2 OS cells were infected with mRFP‐GFP‐LC3 virus, and the images were captured with a confocal microscopy after treatment by siRNA‐Beclin‐1 and/or blue LED for 180 J/cm^2^ and 360 J/cm^2^. Red dots represent autophagosomes and the red and green merged dots represent autolysosomes. (Bar: 50 μm) (D) Bar graph demonstrates that the analysed number of autophagosomes and autolysosomes. E and F, Cells were stained with Trypan Blue, and then counted after treatments of blue LED combined with or without siRNA‐Beclin‐1 transfection. The panels show the number of live cells. G, Schematic picture for blue LED irradiation on EGFR/Beclin‐1‐mediated autophagy in human osteosarcoma. Data are expressed as the mean ± SEM. (Bar: 200 μm) **P < 0.01; ***P < 0.001

Beclin‐1 is an important component of the autophagy‐inducing complex, playing a central role in the autophagy signalling network.^33^ To determine whether the Beclin‐1 inhibition can block blue LED‐mediated induction of autophagy, we treated U‐2 OS cells with blue LED irradiation combined with either transfection of siRNA‐mediated knockdown of Beclin‐1 or negative control (NC) oligos. According to the Western blot and qRT‐PCR results, the expression of Beclin‐1 was significantly down‐regulated after transfection of siRNA‐Beclin‐1 oligos compared with NC group (Figure 6A and 6B). We then evaluated inhibition of Beclin‐1 on blue LED irradiation‐induced autophagy and cell death. As shown in Figures 6C and 6D, siRNA‐Beclin‐1 transfection leads to an abolishment of blue LED irradiation‐induced autophagy. Consistent with our expectations, inhibition of Beclin‐1 resulted in a significant decrease of blue LED irradiation‐induced cell death (Figures 6E and 6F).

## 4 DISCUSSION

In this study, for the first time we found that blue LED irradiation exerted anti‐tumour activities which are evidenced by suppression of cell proliferation, migration and invasion in human U‐2 OS cells. Further study revealed that blue LED irradiated cells displayed increased ROS production and activation of autophagy. Furthermore, we identified EGFR phosphorylation was also significantly inhibited in blue LED irradiated OS cells. The suppressed EGFR caused its binding protein Beclin‐1 release, leading to autophagic cell death. Our study demonstrated that the anti‐tumour action induced by blue LED irradiation was mediated by both ROS and EGFR/Beclin‐1‐mediated autophagy signalling pathway in human OS. This study provides new approach and strategy for the treatment of human OS in clinics.

Currently, autophagy plays essential roles in different cellular processes and has been implicated to be involved in the pathogenesis of a wide variety of diseases, including cardiovascular diseases,^34^, ^35^ atherosclerosis,^36^ neurodegenerative disorders^37^ and more cancers.^7^, ^38^, ^39^ Although several studies have indeed provided data to describe the anti‐tumour effects of blue LED on cancers, its role and in‐depth mechanisms remain unclear. Thus, our study firstly demonstrated the induction of autophagy by blue LED irradiation, leading to suppression of cell proliferation, migration and invasion in human OS. Furthermore, we attempted to identify the mechanisms that autophagy induction by blue LED irradiation was linked to the ROS accumulation and inhibition of EGFR activation (phosphorylation) and EGFR/Beclin‐1 complex formation.

Accumulating evidence suggested that ROS mediated autophagic cell death in multiple cancers.^40^, ^41^ We only determined these effects using ROS inhibitor NAC. It will certainly be interesting to confirm the specific ROS responsible for the induction of LED‐induced autophagy using specific scavengers/inhibitors of different ROS molecules. Moreover, EGFR ectopic activation has been associated with the number of cancers^21^, ^42^, ^43^, ^44^ including lung cancer and glioma. Importantly, Kersting el al. provided 81% patients of OS revealed an expression of EGFR.^45^ Studies have already indicated that EGFR‐mediated RAS/RAF/MEK/ERK signalling pathway plays a critical role in the induction of autophagy in various tumours.^11^, ^46^ Therefore, anti‐EGFR‐based treatments would be therapeutic strategies for cancers. In the current study, we firstly showed interplay between blue LED irradiation and EGFR/Beclin‐1‐mediated autophagy. Besides, inhibition of autophagy using 3‐MA, CQ or siRNA‐Atg7 significantly blocked the anti‐tumour effects of blue LED irradiation on U‐2 OS cells, but the OS cell function was not fully recovered. It might be caused by relatively weaker inhibition efficiency of 3‐MA on autophagy than blue LED irradiation on U‐2 OS cells. On the other hand, blue LED irradiation may also be involved in the specific autophagic elimination of mitochondria, mitophagy that is another important part of autophagy process.

Currently, several EGFR inhibitors have been used in the treatment of cancers, such as tyrosine kinase inhibitors (TKI) (eg erlotinib and gefitinib) and monoclonal antibodies (eg cetuximab and necitumumab).^47^, ^48^ The former bind to the TK domain in the EGFR stopping the EGFR activity. The latter bind to the extracellular component of the EGFR and prevent EGF binding its receptors. In our study, we demonstrated that blue LED irradiation inhibited EGFR phosphorylation and prevented its activation, subsequently leading to depression of cancer cell growth. These motivate the potentials of combination treatments for cancers, but further study needed to test effectiveness and safeness of combination treatments. According to results in Figure 6, our data demonstrated that siRNA‐Beclin‐1 transfection led to a significant decrease of autophagy induced by blue LED irradiation, however, not recover completely on OS cell growth. This implicates that existence of other mechanisms may also be involved in blue LED irradiation‐induced OS cell death.

In line with our results, several studies have also shown that blue LED irradiation caused damage in multiple types of cancer cells such as lymphoid cells,^49^ melanoma^50^ and skin tumours.^51^ In addition to LED light therapy by single colour, a combination of different LED colours has been proven to exert synergistic effects in diseases. Alternated between a wavelength of red (peaked at 630 nm) and infrared radiation (IR, peaked at 870 nm) decreased levels of inflammation in the osteoarthritic joints.^52^ The combination of curcumin with blue LED (peaked at 405 nm) united red LED (peaked at 630 nm or 660 nm) irradiation can attain a higher efficiency of anti‐proliferative and apoptosis‐inducing effects in skin keratinocytes.^53^ Thus, further study will be needed to elucidate the effects of different coloured LED irradiation on OS.

In conclusion, suppressed effects of blue LED irradiation were shown in human OS cells characterized by inhibition of cell proliferation, migration, invasion and induction of cell death via inducing ROS and EGFR/Beclin‐1‐mediated autophagy (Figure 6G). Therefore, our study will provide a better understanding of blue LED irradiation‐mediated autophagy on OS therapy.

## CONFLICT OF INTEREST

The authors indicate no potential conflicts of interest.

## AUTHOR CONTRIBUTIONS

Y.Y., M.Y.H., G.G.Y., Y.W., R.G., H.L, S.T.Y., X.Q.H., G.H.L., W.J.D., T.S.M., M.Q.G., M.X.Y., S.Z.L., Z.H.X. E.I., N.Z and V.P.: performing research; Y.Y., M.Y.H. and G.G.Y.: data analysis; B.Z.C., Y.Y. and L.Y.: study design and writing the manuscript.

## Supporting information

Supplementary Material
