# Supplementary material for: Blue LED causes autophagic cell death in human osteosarcoma by increasing ROS generation and dephosphorylating EGFR
Source: J Cell Mol Med. 2021 May 7;25(11):4962–73. doi: 10.1111/jcmm.16412 (PMC8178260; doi:10.1111/jcmm.16412)
Supplement: Supplementary file 1 — Supplementary Material [file JCMM-25-4962-s001.docx]

**Supplementary data**


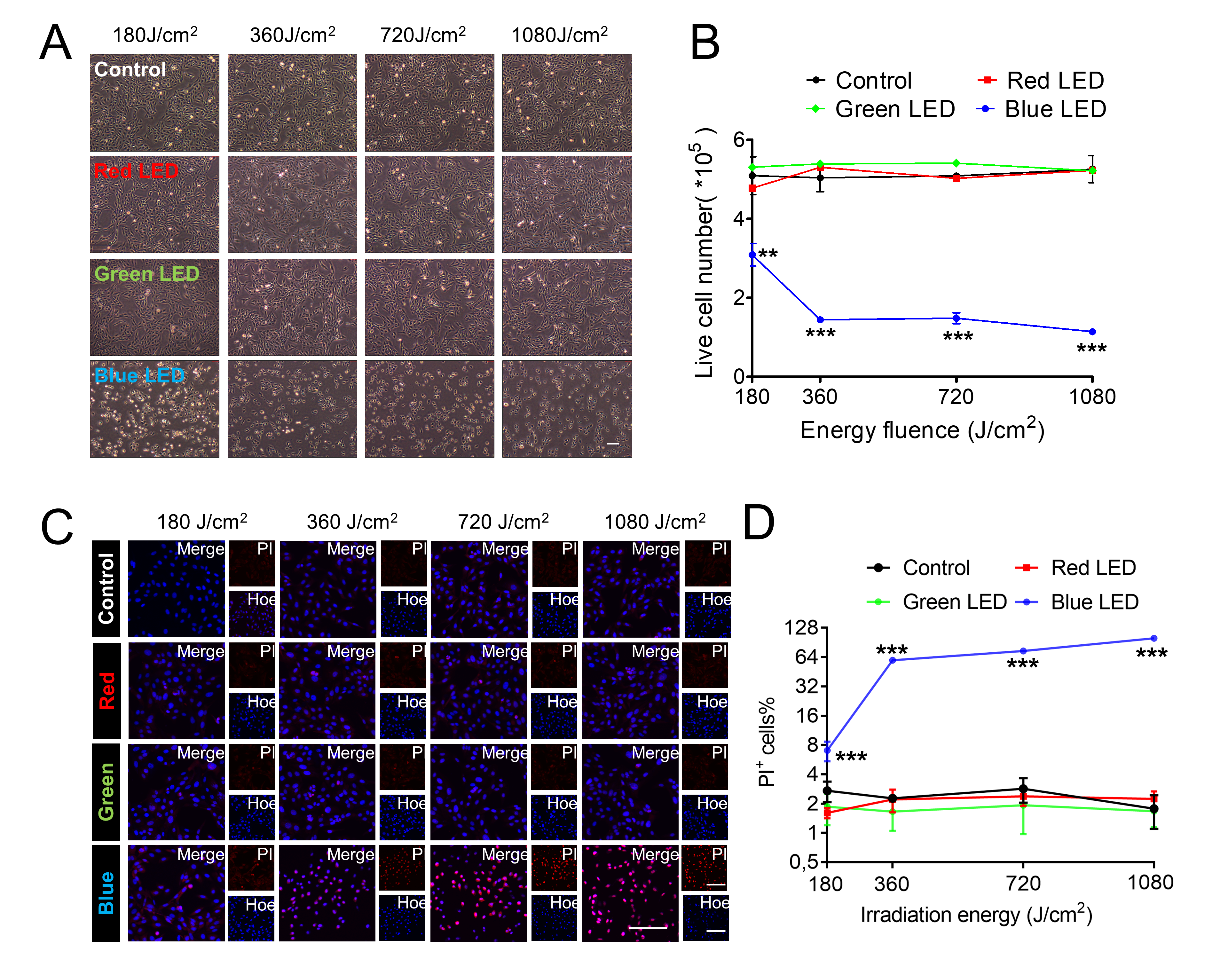


**Supplementary Figure 1. Blue LED irradiation reduces cell growth and induce cell death in U-2 OS cells.** **(A)** U-2 OS cells were irradiated with or without red, green and blue LED lights at a power density of 100 mW/cm^2^ for 180 J/cm^2^, 360 J/cm^2^, 720 J/cm^2^, 1,080 J/cm^2^ respectively. Representative images obtained under a microscope (magnification ×4) 24 hrs after LED exposure. (Bar: 200 μm) **(B)** The panel shows the number of live cells which were counted after stained by Trypan Blue. **(C)** Cell death was determined by PI (red) and Hoechst 33342 (Hoe, blue) staining. (Bar: 100 μm) **(D)** The panel shows the percentages of dead cells (PI positive) in U-2 OS. Data are expressed as the mean ± SEM. **P < 0.01; ***P < 0.001.


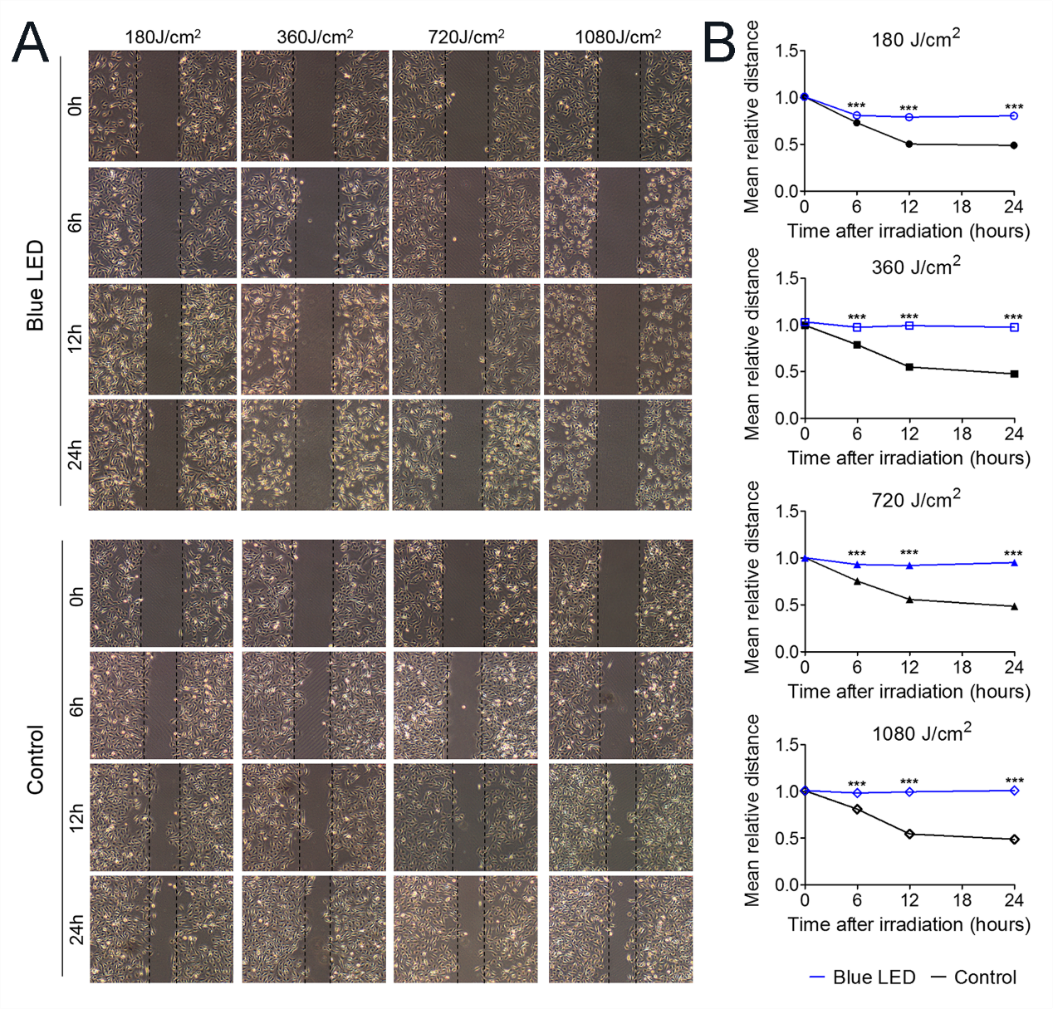


**Supplementary Figure 2. Blue LED irradiation suppresses U-2 OS cell migration** **at shorter time points.** U-2 OS cells were treated with blue LED light for 0 J/cm^2^, 180 J/cm^2^, 360 J/cm^2^, 720 J/cm^2^, 1,080 J/cm^2^ respectively. **(A)** The migration was evaluated by Wound-healing assay. (Bar: 200 μm) **(B)** The mean relative distance of migrated cells. Data are expressed as the mean ± SEM. ***P < 0.001.


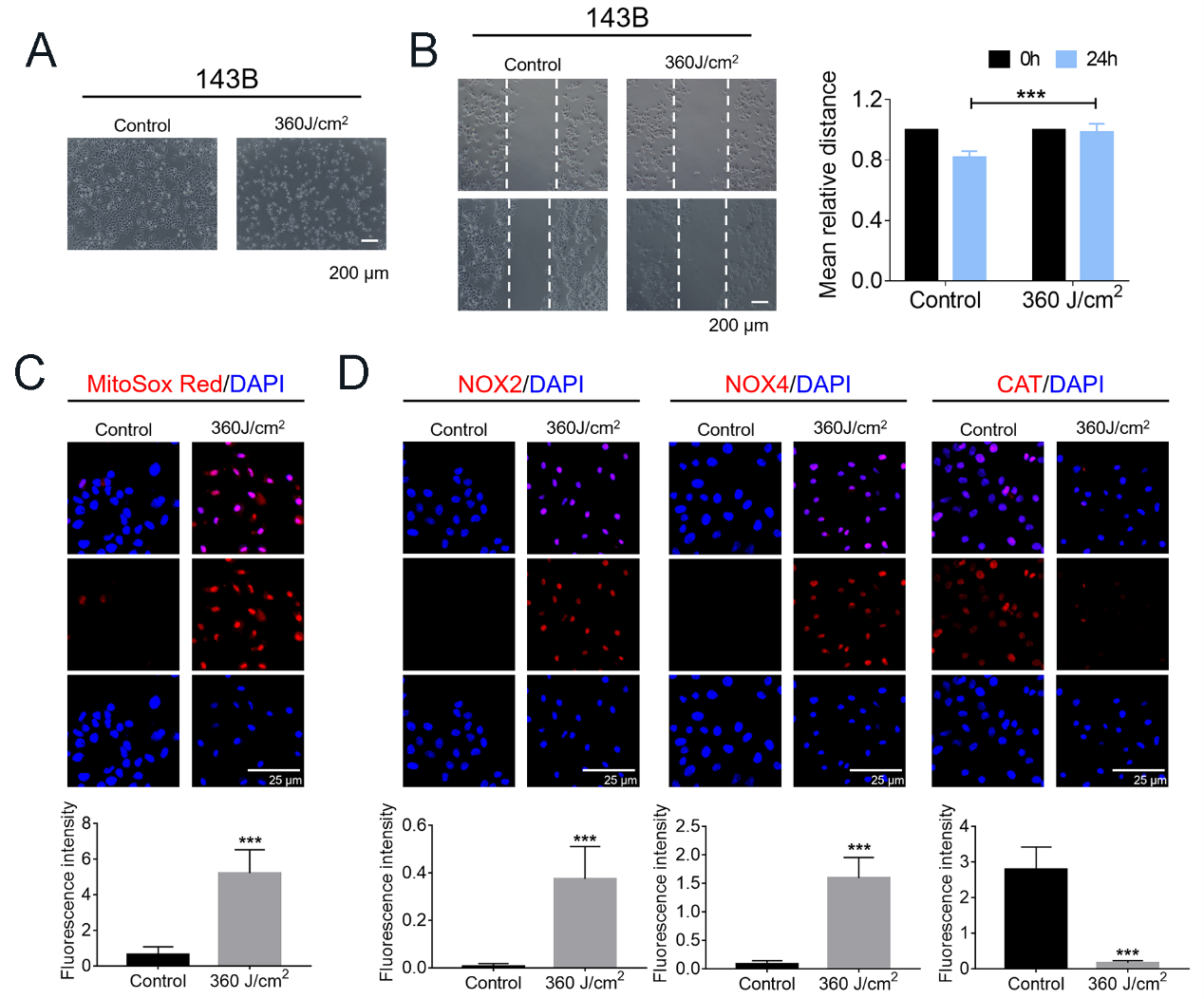
 **Supplementary Figure 3. Blue LED irradiation inhibits 143B cell migration and induce ROS accumulation.** 143B cells were treated with blue LED light for 0 J/cm^2^ and 360 J/cm^2^ respectively. **(A)** Representative images obtained under a microscope (magnification ×4) 24 hrs after LED exposure. (Bar: 200 μm) **(B)** The representative images of migration evaluated by Wound-healing assay. (Bar: 200 μm) The panels show the mean relative distance of migrated cells. **(C)** The level of mitochondrial superoxide in 143B cells detected by MitoSOX Red Indicator staining. (Bar: 50 μm) The panels show the percentage of fluorescence intensity of ROS. **(D)** Immunofluorescence analysis of NOX2, NOX4 and CAT in 143B cells. Nuclei were stained with DAPI. The panels show the percentage of fluorescence intensity of NOX2, NOX4 and CAT. (Bars: 50 μm). Data are expressed as the mean ± SEM. ***P < 0.001.


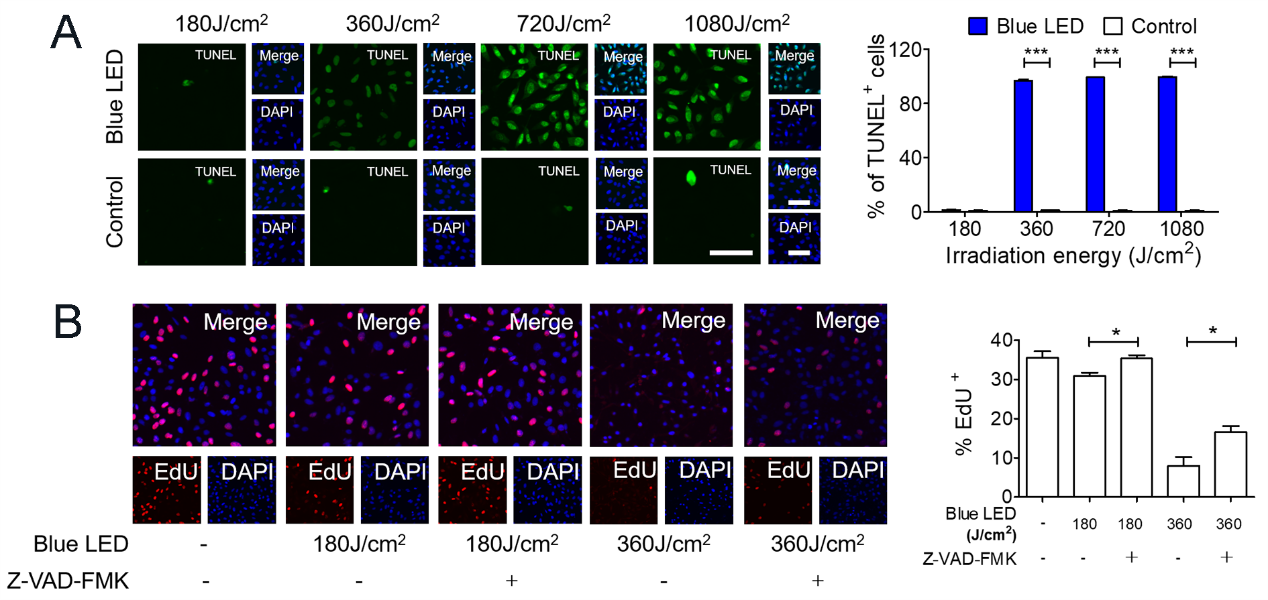


**Supplementary Figure 4. Blue LED irradiation induces cell apoptosis and blocks cell proliferation in U-2 OS cells. (A)** U-2 OS cells were treated with blue LED light for 0 J/cm^2^, 180 J/cm^2^, 360 J/cm^2^, 720 J/cm^2^, 1,080 J/cm^2^ respectively. Cell apoptosis was detected by TUNEL staining 24 hrs after LED exposure. (Bar: 100 μm) The panel shows the percentages of apoptotic cells (TUNEL positive). **(B)** U-2 OS cells were irradiated by blue LED light with or without Z-VAD-FMK treatments. EdU staining evaluated the cell proliferation. The panel shows the percentages of proliferative cells (EdU positive). Data are expressed as the mean ± SEM. *P < 0.05, ***P < 0.001.

**
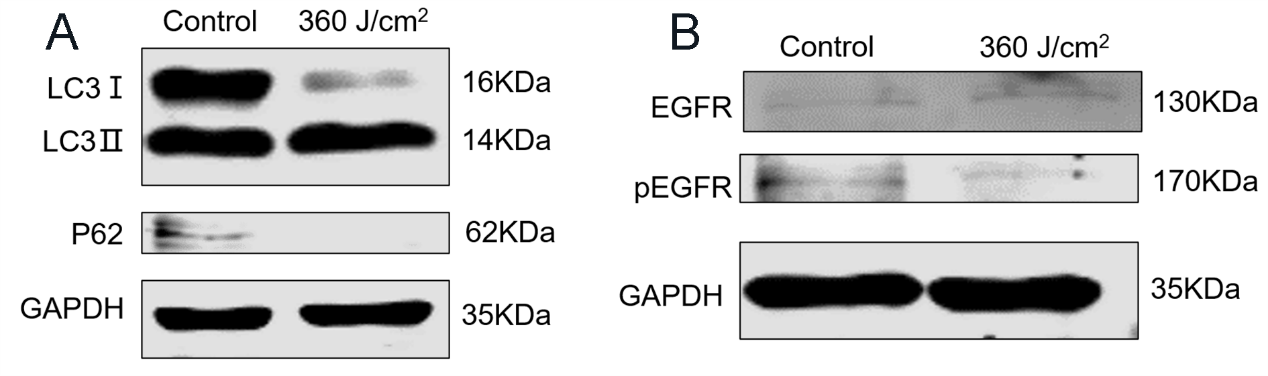
Supplementary Figure 5. Blue LED irradiation induces autophagy and decreases EGFR activation in 143B cells.** 143B cells were treated with blue LED light for 0 J/cm^2^ and 360 J/cm^2^ respectively. (A) Western blot was used to detect the autophagy-associated proteins levels including LC3 and p62. (B) The expression of EGFR and pEGFR detected by Western blot.


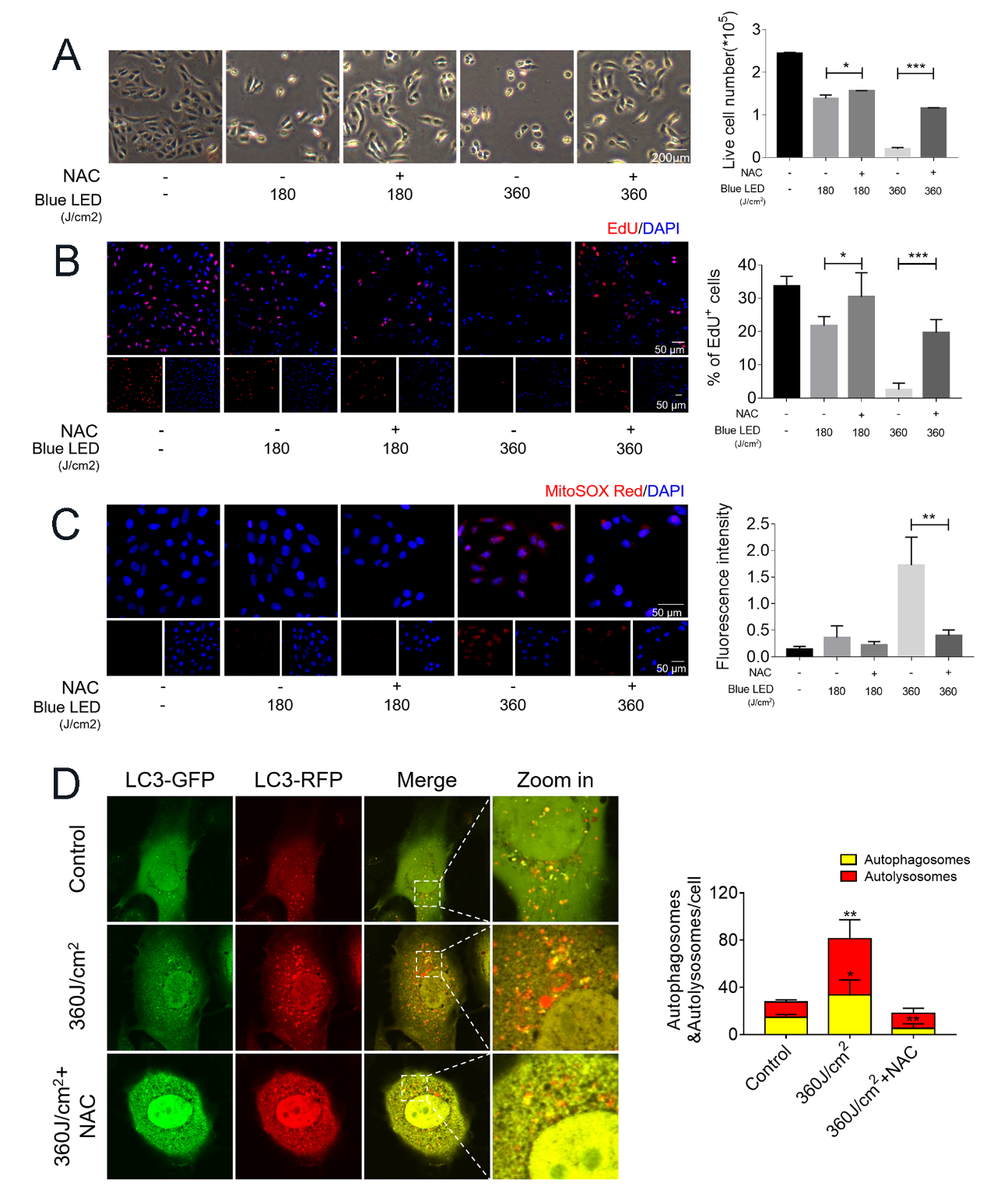


**Supplementary Figure 6. ROS scavenger N-acety-l-cysteine (NAC) recover blue LED irradiation-mediated osteosarcoma cell death and autophagy.** U-2 OS cells treated with blue LED irradiation for 0 J/cm^2^, 180 J/cm^2^, 360 J/cm^2^ with or without NAC (10 mM) respectively. **(A)** Representative images acquired with a microscope (magnification ×4) after irradiation. (Bar: 200 μm). The panels show the number of live cells. **(B)** EdU staining was performed to detect the effect of NAC on the proliferation of U-2 OS cells after exposure to blue LED. (Bar: 50 μm) The panels show percentages of the proliferative cells (EdU positive). **(C)** The level of mitochondrial superoxide was detected by MitoSOX Red Indicator staining. (Bar: 50 μm) The panels show the percentage of fluorescence intensity of ROS. **(D)** Representative cells were examined for ﬂuorescence using confocal microscopy. Yellow puncta: overlapped GFP and RFP images are observed on autophagosomes; red puncta: only RFP ﬂuorescence is observed on autolysosomes. (Bar: 50 μm) Quantiﬁcation of autophagosomes (yellow; GFP^+^ RFP^+^ LC3) and autolysosomes (red; GFP^-^ RFP^+^ LC3) per cell. Data are expressed as the mean ± SEM. *P < 0.05; **P < 0.01; ***P < 0.001.


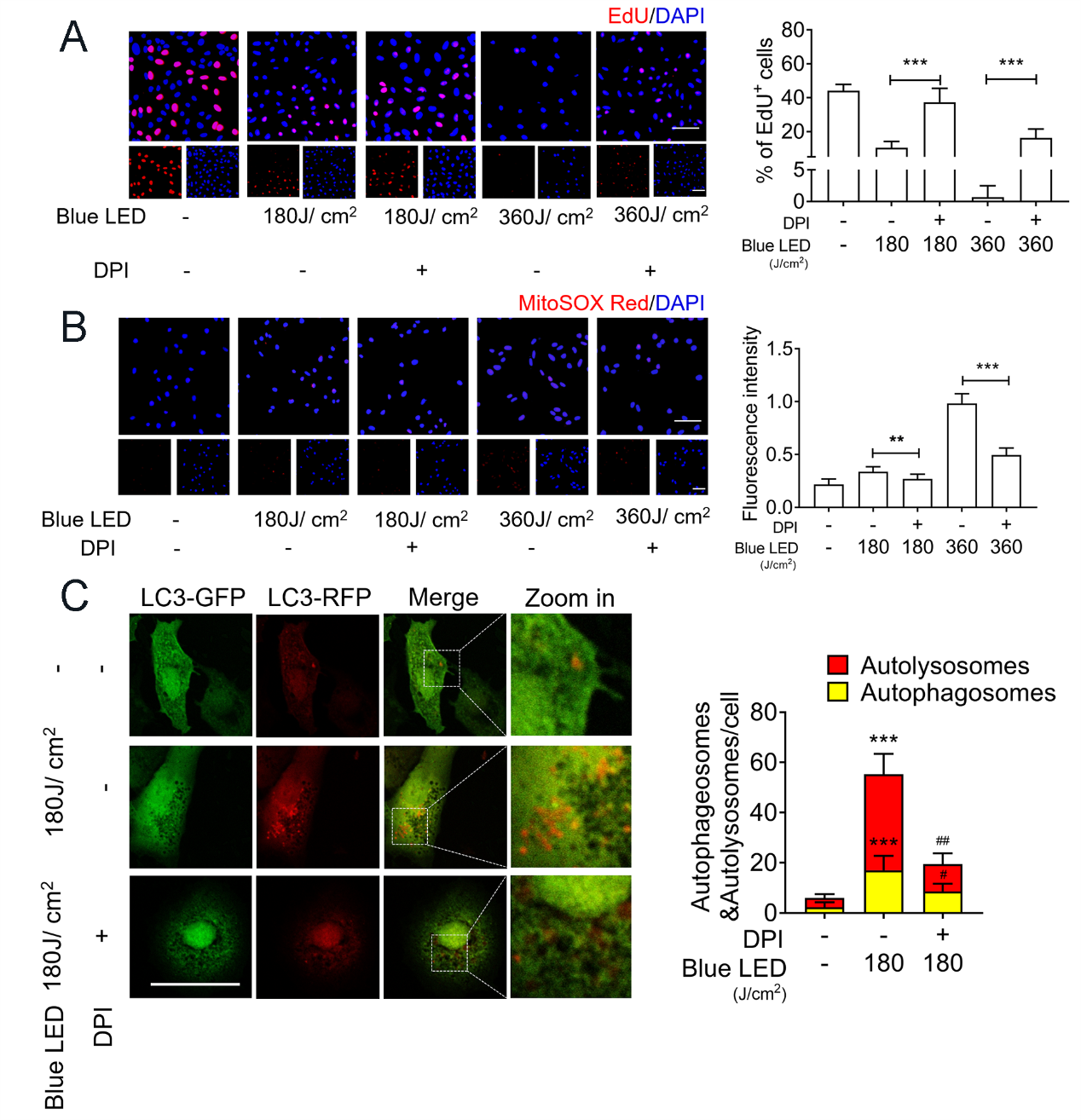


**Supplementary Figure 7. NOX inhibitor** **Diphenyleneiodonium (DPI) reverses blue LED irradiation-mediated osteosarcoma cell death and autophagy.** U-2 OS cells treated with blue LED irradiation for 0 J/cm^2^, 180 J/cm^2^, 360 J/cm^2^ with or without DPI (10 μm) respectively. **(A)** EdU staining was performed to detect the effect of DPI on the proliferation of U-2 OS cells with blue LED irradiation. (Bar: 50 μm) The panels show percentages of the proliferative cells (EdU positive). **(B)** The level of mitochondrial superoxide was detected by MitoSOX Red Indicator staining. (Bar: 50 μm) The panels show the percentage of fluorescence intensity of ROS. **(C)** Representative cells were examined for ﬂuorescence using confocal microscopy. Yellow puncta: overlapped GFP and RFP images are observed on autophagosomes; red puncta: only RFP ﬂuorescence is observed on autolysosomes. (Bar: 50 μm) Quantiﬁcation of autophagosomes (yellow; GFP^+^ RFP^+^ LC3) and autolysosomes (red; GFP^-^ RFP^+^ LC3) per cell. Data are expressed as the mean ± SEM. **P < 0.01; ***P < 0.001(vs. the first group). ^#^P < 0.05; ^##^P < 0.01 (vs. the second group).
